# Manipulation of the Superhydrophobicity of Plasma-Etched Polymer Nanostructures

**DOI:** 10.3390/mi9060304

**Published:** 2018-06-18

**Authors:** Ke Du, Youhua Jiang, Yuyang Liu, Ishan Wathuthanthri, Chang-Hwan Choi

**Affiliations:** 1 Department of Mechanical Engineering, Stevens Institute of Technology, Hoboken, NJ 07030, USA; kdu1@berkeley.edu (K.D.); yjiang10@stevens.edu (Y.J.); tcliuyy@gmail.com (Y.L.); Ishan.Wathuthanthri@ngc.com (I.W.); 2 Department of Chemistry, University of California-Berkeley, Berkeley, CA 94720, USA; 3 Northrop Grumman Mission Systems, Advanced Technology Labs, Linthicum, MD 21090, USA

**Keywords:** polymer, plasma etching, nanostructures, droplet mobility, superhydrophobicity

## Abstract

The manipulation of droplet mobility on a nanotextured surface by oxygen plasma is demonstrated by modulating the modes of hydrophobic coatings and controlling the hierarchy of nanostructures. The spin-coating of polytetrafluoroethylene (PTFE) allows for heterogeneous hydrophobization of the high-aspect-ratio nanostructures and provides the nanostructured surface with “sticky hydrophobicity”, whereas the self-assembled monolayer coating of perfluorodecyltrichlorosilane (FDTS) results in homogeneous hydrophobization and “slippery superhydrophobicity”. While the high droplet adhesion (stickiness) on a nanostructured surface with the spin-coating of PTFE is maintained, the droplet contact angle is enhanced by creating hierarchical nanostructures via the combination of oxygen plasma etching with laser interference lithography to achieve “sticky superhydrophobicity”. Similarly, the droplet mobility on a slippery nanostructured surface with the self-assembled monolayer coating of FDTS is also enhanced by employing the hierarchical nanostructures to achieve “slippery superhydrophobicity” with modulated slipperiness.

## 1. Introduction

The development of bio-inspired nanostructures has drawn great interest in recent years, with a purpose of mimicing the astounding structural complexity presented in nature [1,2,3]. For example, the lotus effect and the petal effect have been widely studied to understand the synergistic effect of the surface energy of different materials and the physical surface morphology [4,5]. For the lotus effect, the hierarchical micro- and nanostructures on a lotus leaf result in a high contact angle (CA) of a water droplet, typically >150° and a low contact angle hysteresis (CAH, the difference between the advancing and receding contact angles), typically <10°. Such a surface with low droplet adhesion has been widely explored for applications including droplet manipulation [6], water-harvesting [7], anti-wetting [8], and anti-fogging surfaces [9]. The CA of a droplet subject to the lotus leaf effect is generally described based on the Cassie-Baxter equation [10]:

(1) $$\cos\theta_{CB}=\phi \cdot \cos\theta_{0}+\phi -1$$

where *θ_CB_* is the CA on a roughed surface, *ϕ* is a ratio of the solid surface area wetted by liquid to the projected area (referred to as a solid fraction), and *θ*_0_ is the CA on a smooth surface with the same material. By fabricating a structured surface with a low solid fraction and depositing a low surface energy material on the surface, *θ_CB_* is increased to have anti-wetting performance.

On the other hand, the hierarchical micro- and nanostructures on a rose petal leaf make a droplet have a high CA but low mobility. Such a surface with high droplet adhesion has potential applications to liquid transportation and analysis [11]. The petal effect can be described using the Cassie-impregnating wetting model [12]. In this case, rather than sitting only on the tips of the solid structures, water can partially penetrate into the grooves in the micro-nanostructures, resulting in a greater solid-liquid contact area compared to a droplet in the pure Cassie-Baxter state.

To realize the petal effect on an artificial surface, two challenges must be overcome: A fabrication method for highly roughened surface must be developed, and the roughened surface should still trap air to some extent to achieve the partial wetting state and thus the high CA despite the increase in the solid-liquid contact area. To increase the surface roughness, advanced nanopatterning techniques such as black silicon [13], metal-assisted chemical etching [14], and anodization [15] have been explored to realize such bio-inspired and high-aspect-ratio nanostructures. To further increase the surface roughness and achieve multiscale hierarchical micro- and nanostructures, a nanostencil lithography technique has also been employed, capable of patterning uniform and high-aspect-ratio nanostructures selectively on top, bottom, or both top and bottom of pre-patterned microstructures [16,17,18,19,20]. However, the minimum feature size of the nanostructures attainable in such a technique is limited by the resolution of the nanostencil. Thus, it remains challenging to pattern hierarchical nanostructures with a feature size of less than 50 nm. Other patterning techniques for hierarchical nanostructures such as dip pen lithography [21], two photon beam polymerization lithography [22], and nanoimprint lithography [23] were also introduced. However, they have limitations to pattern uniform, scalable, high-aspect-ratio, and multiscale hierarchical nanostructures. Recently, a simple one-step maskless plasma etching process has been demonstrated to pattern nanostructures on various polymer surfaces with a high aspect ratio (>20) and a feature size less than 50 nm [24,25]. Many experimental and theoretical models have been developed to explain the self-formation of the high-aspect-ratio nanostructures during the maskless plasma etching process, including crystalline structures [26,27] and impurities [28,29]. For example, many photoresist polymer materials contain metals such as antimony (Sb), which may serve as a hard mask during the etching process [30]. Such nanostructured surfaces of polymers are ideal platform for cell culture [31] and biomarkers immobilization [32]. In addition, the high-aspect-ratio polymer nanostructures allow the design and fabrication of durable superhydrophobic and superamphiphobic surfaces with a great control of wettability and light reflectance [33,34,35,36]. Integrating with laser interference lithography, the fabrication of uniform and large-area pillar-on-pore and pillar-on-pillar hierarchical nanostructures on various polymer surfaces have also been demonstrated with a good control of the solid surface area [37].

Another challenge in mimicking natural surface systems with modulated droplet adhesion is how to effectively control the droplet mobility on an artificial surface. The droplet mobility is fundamentally determined by two factors: The intrinsic droplet-solid adhesion force, which is characterized by the advancing and receding CA on a smooth surface, and the microscopic interaction between the liquid and the solid structures, which is essentially determined by the configuration of a three-phase contact line and its dynamics [38,39,40]. The first factor can be controlled by modifying the surface energy through methods such as using chemical coating, while the second factor can be controlled by physically regulating the morphological dimensions of the solid surface. However, the control of surface chemistry and morphological dimension are challenging, especially at nanoscale [41], which limits the scope of natural systems that can be mimicked.

In this paper, we show that droplet mobility on a nanotextured polymer surface obtained by the maskless plasma etching process using self-masking effects can effectively be controlled by regulating the hydrophobic coating methods and furthermore the structural hierarchy. In particular, a spin-coating of polytetrafluoroethylene (PTFE) is employed on the nanostructured polymer surface by the oxygen plasma etching to achieve the Cassie-impregnating wetting regime with a high CA but low droplet mobility. On the other hand, a self-assembled monolayer coating of 1H,1H,2H,2H-perfluorodecyltriethoxysilane (FDTS) is employed on the nanostructured polymer surface by the oxygen plasma etching to achieve the Cassie-Baxter wetting state with a high CA and high droplet mobility. We also demonstrate the increase in the droplet CA in the Cassie-impregnating wetting regime while keeping the low droplet mobility by employing hierarchical nanostructures via the combination of nanolithography (laser interference lithography) to the oxygen plasma etching. As for the droplet in the Cassie-Baxter wetting state, we also demonstrate the enhancement of droplet mobility by reducing the solid-droplet contact area controlled by the plasma etching time.

## 2. Materials and Methods

**Figure 1:**
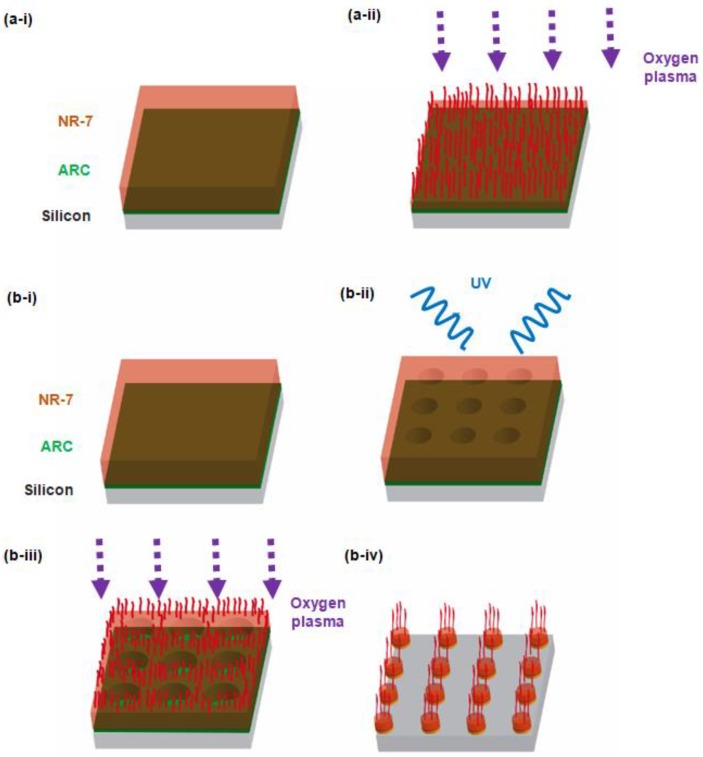
(**a**) Schematic of the fabrication of monotonous polymer nanostructures on a planar surface: (**a**-**i**) Spin-coating of anti-reflective coating (ARC) and NR-7 polymer on a polished silicon substrate; (**a**-**ii**) Patterning of polymer nanostructures via maskless oxygen plasma etching. (**b**) Schematic of the fabrication of hierarchical nanostructures: (**b**-**i**) Spin-coating of ARC and NR-7 polymer; (**b**-**ii**) Laser interference lithography to form a square array of a submicron hole pattern onto the NR-7 polymer layer to serve as base structures; (**b**-**iii**): Oxygen plasma etching; (**b**-**iv**): Pillar-on-pillar hierarchical nanostructures of NR-7 polymer.

The methods of patterning high-aspect-ratio nanostructures and hierarchical nanostructures of polymer on the basis of the maskless plasma etching process are presented in Figure 1. While the details were already reported elsewhere [37], the brief procedures are in the following. First, a polished silicon wafer was cleaned by piranha solution (a mixture of concentrated sulfuric acid with hydrogen peroxide in a ratio of 3:1 in volume), where a polymer film is deposited. For the polymer layer, an anti-reflective coating (ARC, XHRiC 16, Brewer Science) was spun on the silicon wafer to a thickness of 200 nm (2000 rpm for 10 s) followed by baking on a hotplate at 175 °C for 1 min. Then, NR-7 negative photoresist polymer (Futurrex Inc.) was spun on the ARC to a thickness of 1.5 µm, followed by the soft-bake on a hotplate at 150 °C for 1 min. The NR-7 photoresist layer serves as the polymer to be patterned and etched (Figure 1a-i), while the ARC is applied to minimize the reflection of the light in the nanolithography process employed for the hierarchical nanopatterning (Figure 1b-i). In the case of the fabrication of monotonous nanostructures on the planar polymer surface (Figure 1a), a reactive ion etching (RIE) process (Phantom III, Trion Technology) was directly applied. The RIE power was set at 50 W with the DC bias voltage of 300 V and the oxygen gas flow rate and the chamber pressure were set at 30 sccm and 100 mTorr, respectively. During the etching process, the temperature of the silicon wafer was maintained at 25 °C. Due to the self-masking effects, pillared polymer nanostructures in the scale (diameter) of sub-100 nm are conveniently obtained without using any artificial etch masks. For the case of NR-7 photoresist polymer material employed in this study, the self-masking effect should be caused by the combination of the antimony element possessed by the NR-7 polymer material itself and the co-deposition of a silicon element elsewhere (e.g., sputtered from the etching chamber wall or the silicon substrate during the plasma etching) [37]. The aspect ratio of the polymer nanostructures can be regulated by controlling the etching time. The dimensions and aspect ratios of the fabricated nanostructures were measured bythe scanning electron microscope (SEM) images using the SmartSEM software (ZEISS). In the case of the fabrication of multiscale hierarchical nanostructures (Figure 1b), laser interference lithography of a Lloyd-mirror configuration [42] was first used to create submicron structures of polymer (Figure 1b-ii), where the smaller (sub-100 nm) nanostructures would additionally be created by the oxygen plasma etching to allow the hierarchy (Figure 1b-iii,1b-iv). For the submicron structures that serve the first layer for the hierarchy, a square array of a hole pattern with a pattern periodicity of 935 nm and the hole diameter of ~500 nm was created via the laser interference lithography (Figure 1b-ii). Then, the oxygen plasma etching was applied onto the polymer layer featured with the submicron hole pattern with the modulation of the etching time (180, 300, 420, and 600 s in this study) to form the various types of hierarchical nanostructures of the polymer. While the secondary sub-100 nm polymer structures with a higher aspect ratio is generally achieved on top of the first layer of the polymer with the increase in the plasma etching time (Figure 1b-iii), the first layer with a hole pattern becomes transformed to have a pillar pattern due to the isotropic etching characteristics of the oxygen plasma etching process (Figure 1b-iv) [37].

To modulate the hydrophobicity of the polymer nanostructures, two different coating methods were applied onto the plasma-etched polymer surfaces. The first method employed was a spin-coating of PTFE Teflon^®^ AF1600 (DuPont, Wilmington, DE, USA), while the second one was a self-assembled monolayer (SAM) coating of FDTS (1H,1H,2H,2H-perfluorodecyltrichlorosilane, Alfa Inc.). For the spin-coating of PTFE, the coating thickness was controlled to be less than 10 nm by regulating the spin speed and time (~3000 rpm for 40 s in this study) of 0.2 wt % PTFE Teflon^®^ AF1600 solution (DuPont, Wilmington, DE, USA). For the SAM coating of FDTS, the plasma-etched polymer surface was immersed in 1 mM FDTS solution in iso-octane in N_2_drybox at room temperature for a couple of hours, which created a uniform monolayer film thickness of ∼2 nm [43]. Then, the surface was rinsed by 2-propanol and water for 5 min, respectively, and dried in air for 1 day before the testing of surface superhydrophobicity.

For the characterization of the superhydrophobicity of the plasma-etched polymer surfaces with the two different types of the hydrophobic coatings, the static and dynamic (advancing and receding) contact angles (CAs) of a sessile droplet (∼3 μL) of deionized water were measured on each surface at a room condition right after the coatings. A goniometer system (Model 500, Rame-hart) was used for the measurement of CAs, capable of automatic tilting of the sample stage. For the measurement of static CAs, a water droplet was deposited on the fabricated surface and the static CA was determined when the droplet became static (e.g., ~10 s after the deposition). For the measurement of dynamic CAs (i.e., advancing and receding CAs) of the water droplet, the CAH (the difference between advancing and receding CAs) and the sliding angle (SA, the inclination angle of the surface at which a droplet slides) were measured while the sample stage was gradually tilted from 0 to 90° at the rate of 1° per second. The advancing and receding CAs were measured at the moment when a droplet started to slide. The average and standard deviation values of the CA, CAH, and SA were obtained by more than three measurements conducted at different locations of each specimen. In the case of the complete pinning where a droplet does not slide even when the surface is tilted at 90°, the CAH and SA cannot be determined and thus the surface is referred to as “sticky”.

## 3. Results and Discussion

### 3.1. Superhydrophobicity of the Plasma-Etched Polymer Surface with Monotonous Nanostructures

**Figure 2:**
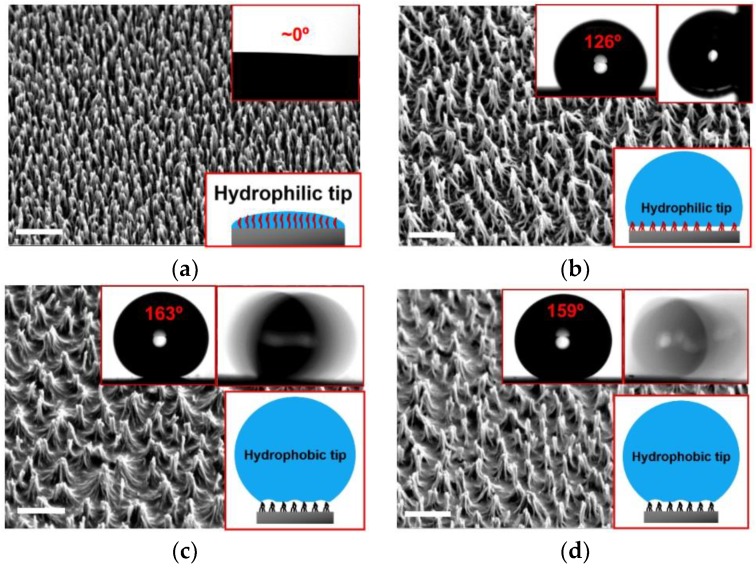
SEM images of the polymer nanostructures obtained with the oxygen plasma etching for 420 s, followed by different types of hydrophobic coatings. (**a**) Initial polymer nanostructures after the plasma etching (i.e., no hydrophobic coating). (**b**) Spin coating with PTFE. (**c**) SAM coating of FDTS in a dipping mode. (**d**) Spin coating with PTFE, followed by the SAM coating of FDTS in a dipping mode. The scale bar in each SEM image represents 1 µm. Insets in each image show the measurements of CA and CAH/SA, as well as the schematics of the wetting states.

Figure 2a shows the SEM image of the plasma-etched polymer surface (NR-7) for 420 s. The maskless one-step plasma etching process can effectively create sub-100 nm pillared nanostructures of various polymers with a high aspect ratio, employing the self-masking effect [25,37]. The height of the nanostructures generally increases with the etching time, while the diameter almost remains the same. After the oxygen plasma etching for 420 s, the pillared nanostructures of NR-7 polymer have the tip (circular pillar top) diameter of ~40 nm, the aspect ratio of ~20 (i.e., the height close to 1 μm), and the average pattern periodicity of ~90 nm. The results are reproducible with the standard deviations of ~10 nm for the tip diameter and ~50 nm for height [37]. The oxygen plasma etching makes the polymer surface highly hydrophilic due to the OH- group introduced by the oxygen plasma. The dense-array high-aspect-ratio nanostructures enhance the hydrophilicity furthermore due to the significantly increased surface roughness, following the Wenzel model [44]. Thus, the initial polymer surface with the oxygen plasma etching shows the complete wetting with the CA of nearly 0°, where the CAH and SA of a droplet cannot practically be measured.

Figure 2b shows the SEM image of the plasma-etched polymer surface, followed by the spin-coating of PTFE. Due to the capillary force (referred to as a nanocarpet effect [45]) of the PTFE solution during the spin coating, several nanopillar structures became merged to form conical nanostructures of a larger size (tip diameter (*d*): ~100 nm, height (*h*): ~1 μm, aspect-ratio (*h*/*d*): ~10, and pattern periodicity (*λ*): ~500 nm). According to the new structural geometry and dimensions, the expected CA of a sessile droplet of water on such a surface in a Cassie-Baxter wetting state (Equation (1)) should be ~170° with a solid fraction (*ϕ*) of 0.03 (*ϕ* = *πd*^2^/(4*λ*^2^)) and the CA of a smooth PTFE surface (*θ*_0_) is ~110°. However, the CA of a sessile droplet of water measured on the nanostructured polymer surface with the spin-coating of PTFE was 126 ± 3°. Moreover, the droplet was completely pinned on the surface so that it did not slide down even when the surface was tilted vertically (90°). It indicates that the PTFE coating cannot support the pure Cassie-Baxter wetting state. According to the Wenzel equation (cos*θ_W_* = *r*cos*θ*_0_) [44], where a droplet should completely wet the nanotextured surface with a roughness factor (*r*, entire surface area normalized by the projected area, which is estimated as *r* = 1 + *πdh*/*λ*^2^) of ~2.3, the expected CA on the PTFE-coated surface corresponding to a pure Wenzel state (*θ_W_*) should be ~140°, which is still significantly greater than the measured CA (126 ± 3°). Thus, it suggests that the wetting state should correspond to the Cassie-impregnating wetting state, where some gas pockets should still be trapped underneath the droplet while the water contact angle of the wetted surface (e.g., tips) should be much less than that on a PTFE-coated surface, as illustrated as a schematic (inset) in Figure 2b. In other words, the sticky hydrophobicity (i.e., 90°< CA < 150° with significant pinning) is attributed to the non-uniform hydrophobization of PTFE resulted from the nature of the spin-coating process employed. The coating thickness and uniformity in spin coating is very sensitive to the spin speed (i.e., centrifugal force), the viscosity (e.g., concentration) of coating solution, and roughness of the surface [46,47]. The PTFE is likely to get deposited only on the bottom areas of the nanostructures, while the top areas of the nanostructures effectively remain uncoated, with the relatively high spin-speed (3000 rpm) and the relatively low concentration of the PTFE solution (0.2 wt %). Then, the non-uniform coating of PTFE results in a chemical hierarchy to the nanostructures composed of hydrophilic tops (or tips) and hydrophobic bases. Thus, a water droplet cannot completely wet the nanostructured surface due to the hydrophobic bases where air should still be trapped. Such structures then can maintain hydrophobicity (relatively high CA), while they also allow significant pinning and stickiness (adhesivity) of the water droplet due to the increase in the wetted solid surface area by the hydrophilic tips of the nanostructures.

On the contrary, the plasma-etched polymer surface with the SAM coating of FDTS shows totally different behaviors from the spin-coated surface with PTFE. As shown in Figure 2c, the CA of a sessile water droplet on the FDTS-coated surface was 163 ± 3°. The water droplet also easily slid on the surface, with a negligible SA (<5°) and a relatively low CAH (~20°). Considering that the CA of a FDTS coating applied on a smooth surface is ~110°, the high CA with low SA/CAH on the FDTS-coated surface indicates that the surface fully supports the Cassie-Baxter wetting state, as illustrated in the schematic (inset) in Figure 2c. Compared to the spin-coating of PTFE where the inertia effect (i.e., centrifugal force) significantly influence the uniformity of the hydrophobic coating, the FDTS coating is based on the SAM coating in a dipping mode which guarantees the monolayer-thick, uniform and strong hydrophobic coating onto the hydrophilic polymer surface. Thus, the nanostructured polymer surface is fully hydrophobized by the FDTS monolayer with no chemical hierarchy or heterogeneity.

To confirm the non-uniform characteristic of the spin-coating of PTFE, the PTFE-coated surface was additionally coated by the SAM coating of FDTS and the surface hydrophobicity was further examined. As shown in Figure 2d, the CA, CAH, and SA on the surface double-coated by PTFE and FDTS are similar to those coated only by FDTS. It indicates that the surface now possesses uniform hydrophobicity, where the hydrophilic top portions of the initial PTFE-coated surface become hydrophobic with the FDTS coating. These results show that the surface hydrophobicity and droplet mobility (slippery vs sticky) on the nanostructured polymer surfaces via oxygen plasma etching can effectively be regulated by adopting different coating methods.

### 3.2. Superhydrophobicity of the Plasma-Etched Polymer Surface with Hierarchical Nanostructures

Whereas the SAM-coating of FDTS on the plasma-etched polymer surface allowed us to create a “slippery superhydrophobic” surface (i.e., CA > 150° with little pinning), the spin-coating of PTFE resulted in a “sticky hydrophobic” surface (i.e., 90°< CA < 150° with complete pinning). In many applications such as localized reaction, no-lost transportation of droplets, preparation of protein micro-arrays, and in-situ detection of R6G molecules in aqueous and organic liquids [48,49,50,51], it is desirable to have a “sticky superhydrophobic” surface (i.e., CA > 150° with significant or complete pinning). To further enhance the surface hydrophobicity (i.e., CA) while maintaining the high droplet pinning and retention to realize such sticky superhydrophobic surfaces, there are two pathways: Modifying the intrinsic hydrophobicity of the surface and tailoring the effective contact area between the solid surface and a droplet (i.e., solid fraction). Since the high droplet retention (stickiness) was already achieved by the non-uniform characteristics of the spin-coating of PTFE, any further modulation of the surface hydrophobicity is not desirable. Thus, in order to increase only the CA, without sacrificing the high droplet retention property, the modulation of the solid fraction should be the proper pathway. Since the geometric dimensions such as the diameter and periodicity of the pillared nanostructures obtained by the oxygen plasma etching remain almost constant regardless of the etching times and RIE powers [37]. The solid fraction cannot effectively be regulated only by the means of the maskless plasma etching process. Therefore, in this study, we have also examined the combination of the laser interference lithography with the plasma etching process in order to design hierarchical nanostructures which can regulate the solid fraction of the nanostructured polymer surface. As illustrated in Figure 1b-ii, a square array of submicron-scale hole patterns (pattern periodicity of 935 nm and the hole diameter of ~500 nm) of the polymer were prepared by using a laser interference lithography. Then, the sub-50 nm-scale pillared nanostructures were hierarchically patterned on top of the surface by using the oxygen plasma etching with modulated etching time. Then, the hydrophobicity of the polymer surface with the hierarchical nanostructures was examined with both the spin-coating of PTFE and the SAM-coating of FDTS for comparison.

**Figure 3:**
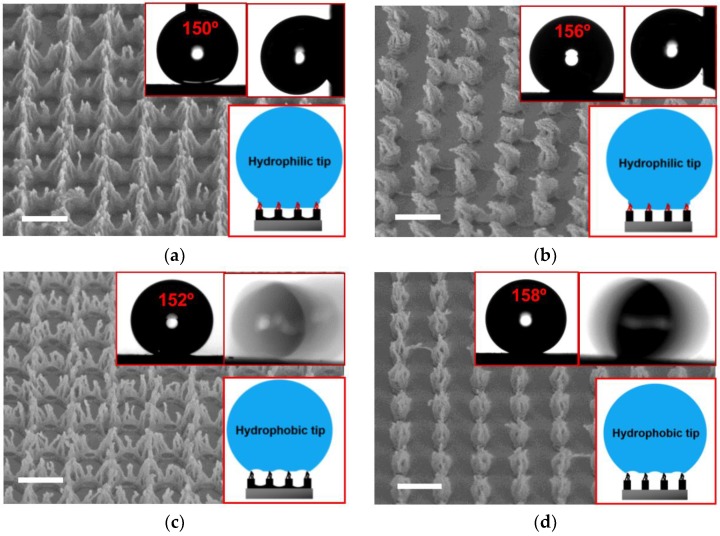
SEM images of hierarchical polymer nanostructures obtained with the oxygen plasma etching for 180 s (**a**,**c**) and 420 s (**b**,**d**), followed by the spin-coating of PTFE (**a**,**b**) and the SAM-coating of FDTS (**c**,**d**), respectively. The scale bar in each SEM image represents 1 µm. Insets in each image show the measurements of CA and CAH/SA, as well as the schematics of the wetting states.

Figure 3a,b show the hierarchical nanostructures of the NR-7 polymer obtained with the plasma etching for 180 and 420 s on top of the submicron-scale hole patterns, respectively, both with the spin-coating of PTFE. With the relatively short etching time (180 s), as illustrated in Figure 1b-iii, the initial hole pattern remains despite the thinned pore walls and the formation of the sub-50 nm pillar structures on the top surface (Figure 3a). The longer etching time (420 s) resulted in more significant over-etching of the hole pattern so that the initial hole pattern completely transformed to a pillar pattern, while the sub-50 nm nanostructures were hierarchically formed on top of the pillar pattern (Figure 3b), as illustrated in Figure 1b-iv. In the case of the pillar structures made with the longer etching time (Figure 3b), the top nanostructures were slightly bent after the spin-coating of PTFE, which was due to the centrifugal force during the spinning. With the spin-coating of PTFE on the polymer surface with the hierarchical nanostructures, the CAs significantly increased to show superhydrophobicity (i.e., CA > 150°), which is due to the significant decrease in the solid fraction by the large (submicron) pattern periodicity of the base structures formed by the laser interference lithography. The over-etching of the initial hole pattern made the surface have the lower solid fraction with pillared morphology so that the CA on the surface with the plasma etching for 420 s is higher (~156°, Figure 3b) than that for 180 s (~150°, Figure 3a). Meanwhile, both surfaces still show the same significant pinning so that the droplet has complete pinning on the surfaces even being vertically tilted (90°). It further supports that the spin-coating of PTFE causes the non-uniform hydrophobization so that the top parts (e.g., tips) of the hierarchical nanostructures retain initial hydrophilicity with little coating of PTFE, as illustrated in the schematic (inset) in Figure 3a,b. Thus, despite the distinct surface morphologies, both surfaces support the Cassie-impregnating wetting state, showing the sticky superhydrophobicity (i.e., CA > 150° with complete pinning).

In contrast, Figure 3c,d show the hierarchical nanostructures of the polymer obtained with the same plasma etching time (180 and 420 s, respectively), but with the SAM-coating of FDTS. The surface morphology of the hierarchical nanostructures with the SAM-coating of FDTS is around the same as that with the spin-coating of PTFE, while there was no deformation (bending) of the top nanostructures in the case of the SAM-coating of FDTS done in a dipping mode. Thus, the CAs of both surfaces also show superhydrophobicity (i.e., CA > 150°), similar to those coated with PTFE. Agreeing with the case with the PTFE coating, the more sharpened hierarchical nanostructures with the elongated etching time result in the higher CA (~158°, Figure 3d) than that with less sharpened hierarchical nanostructures (152 ± 2°, Figure 3c), due to the more significant decrease in the solid fraction. Meanwhile, compared to the monotonous nanostructures (163 ± 3°, Figure 2c), the CAs of both surfaces are not increased. That is because the effective pattern periodicity of the monotonous nanostructures was increased by the capillary effect (i.e., aggregation) after the FDTS coating so that the solid fraction of the polymer surface with the monotonous nanostructures was comparable to that of the polymer surface with hierarchical nanostructures after the FDTS coating. It should also be noted that the hierarchical nanostructures with the SAM-coating of FDTS showed the similar slipperiness with negligible SA (<5°), regardless of the hierarchy. It also indicates that the hierarchically nanostructured polymer surface is also homogeneously coated by the SAM-coating of FDTS so that it supports the Cassie-Baxter wetting state as illustrated in the schematics (insets) in Figure 3c,d. Meanwhile, the more sharpened hierarchical nanostructures with the elongated etching time showed the lower CAH (~7°, Figure 3d) than that with less sharpened hierarchical nanostructures (~15°, Figure 3c), which is also due to the more significant decrease in the solid fraction.

**Figure 4:**
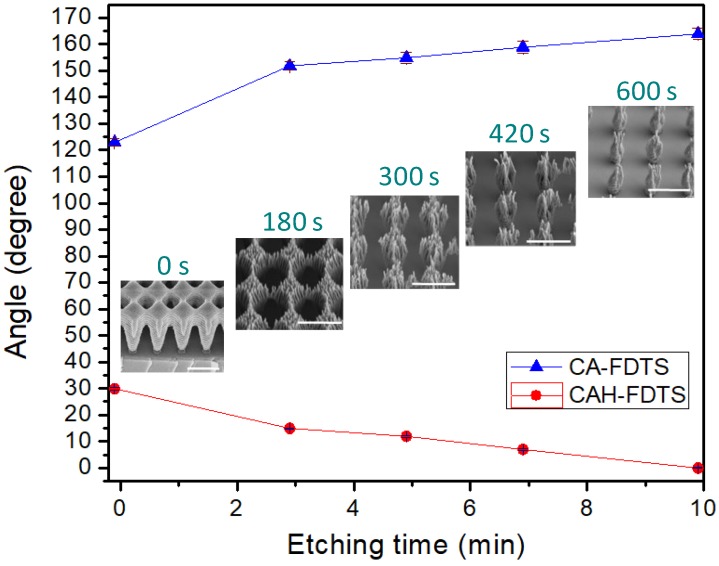
Water droplet CA and CAH measurement for FDTS-coated hierarchically-nanostructured surfaces with plasma etching time ranging from 0 to 10 min. The surface at 0 min refers to the NR-7 surface patterned with laser interference lithography but without oxygen plasma etching. The inserted images are the SEM images of the nanostructures obtained with the different etching times before the coating of FDTS. The scale bar in each inset represents 1 µm.

In the case of the polymer surface with hierarchical nanostructures coated with FDTS, the CA and CAH were further controllable with the modulation of etching time (i.e., the regulation of the solid fraction). Figure 4 shows the variations of the CA and CAH of a sessile droplet of water on the polymer surface with hierarchical nanostructures with respect to the different etching time followed by the SAM-coating of FDTS. It should be noted that the sliding angles are not shown with respect to the etching time in Figure 4 because the sliding angles are already negligibly low so that the noticeable trend was not measurable with respect to the structural hierarchy. The initial surface only with a submicron hole pattern (etching time: 0 s) shows the CA of ~123° and the CAH of ~30°, coated with the FDTS. With the increase in the etching time, which transformed the initial submicron hole pattern to the slender pillar pattern with hierarchical nanostructures, the CA gradually increased (as high as ~162°) while the CAH gradually decreased (as low as ~3°), making the surface with the FDTS coating a more slippery superhydrophobic surface. In particular, the CA increased due to the decrease in the solid fraction of the hierarchically-nanostructured surface with the elongated etching time [52]. Meanwhile, the CAH decreased more fundamentally due to the decrease in the effective length of the three-phase contact line sliding on the top of nanostructures and the distortion of liquid-gas interface in the vicinity of the contact line [53,54,55]. The reduction of the effective contact area between the solid surface and a droplet (i.e., solid fraction) on the hierarchically-pillared nanostructures leads to the decrease in both factors and thus the CAH.

## 4. Conclusions

In this study, we have introduced a simple pathway to regulate droplet mobility on the oxygen-plasma-etched nanostructured polymer surfaces with the different types of hydrophobic coatings including the spin-coating of PTFE and the SAM-coating of FDTS in a dipping mode. A water droplet shows low retention with high CA (>150°) (i.e., slippery superhydrophobicity) on the plasma-etched nanostructured polymer surface with the SAM-coating of FDTS. In contrast, a water droplet shows a higher retention with lower CA (<150°) (i.e., sticky hydrophobicity) on the plasma-etched nanostructured polymer surface with the spin-coating of PTFE. This contrast is because the tips of the oxygen-plasma-etched hydrophilic nanostructures cannot be completely hydrophobized with PTFE in the spin-coating mode due to the significant centrifugal force, while they are homogeneously hydrophobized with FDTS in the SAM-coating in a dipping mode. The reduction of the effective contact area between a droplet and the polymer surface (i.e., sold fraction) by employing the multiscale hierarchy of the structural morphology, enabled by the combination of the laser interference lithography and the maskless plasma etching, leads to the significant increase in CA on the PTFE-coated surface while the high droplet retention is maintained, allowing the surface to have sticky superhydrophobicity and mimic the rose-petal effect. The multiscale hierarchy also helps the FDTS-coated surface to have the slippery superhydrophobicity with modulated CA and droplet mobility (CAH/SA), mimicking the lotus effect. The efficient controllability of the superhydrophobicity and droplet mobility on polymer surfaces, enabled by the maskless plasma-based etching process assisted by the combination with conventional lithography and the modulation of hydrophobic coating process, will be of great significance in many applications, such as slippery superhydrophobicity for anti-icing surfaces [56], enhanced condensation [57], droplet manipulation [58], and fog harvesting [59], and sticky superhydrophobicity for no-lost transportation of droplets [49], preparation of protein micro-array [50], in-situ detection [51], and localized reactions [60].
